# Ecology and seasonality of sandflies and potential reservoirs of cutaneous leishmaniasis in Ochollo, a hotspot in southern Ethiopia

**DOI:** 10.1371/journal.pntd.0007667

**Published:** 2019-08-19

**Authors:** Myrthe Pareyn, Emma Van den Bosch, Nigatu Girma, Natalie van Houtte, Stefan Van Dongen, Gert Van der Auwera, Fekadu Massebo, Simon Shibru, Herwig Leirs

**Affiliations:** 1 Evolutionary Ecology group, University of Antwerp, Antwerp, Belgium; 2 Biology Department, Arba Minch University, Arba Minch, Ethiopia; 3 Department of Biomedical Sciences, Institute of Tropical Medicine, Antwerp, Belgium; Saudi Ministry of Health, SAUDI ARABIA

## Abstract

**Background:**

Ochollo is a village in southern Ethiopia burdened with cutaneous leishmaniasis (CL), where *Phlebotomus pedif*er is the only vector for *Leishmania aethiopica* and hyraxes are confirmed reservoir hosts. A detailed description of the different players of transmission, and the ecology and seasonality of the vector needs to be established in order to accomplish efficient control programs.

**Methods and findings:**

Between March 2017 and February 2018, a monthly sandfly collection was carried out in different habitats and records of temperature and humidity were taken. Rodents and hyraxes were trapped in the dry and wet season. All samples were screened for *Leishmania* kinetoplast DNA (kDNA). Positive samples were further processed for determination of the *Leishmania* species and the species of the sandfly/small mammal that was found infected. Additionally, the species of 400 sandfly specimens from different habitats and seasons was identified.

17,190 *Sergentomyia* and *Phlebotomus* sandflies were caught and showed an overall kDNA prevalence of 2.6%, all were *L*. *aethiopica* infections only found in *P*. *pedifer*. The overall sandfly and *P*. *pedifer* abundance peaked in the dry season and was negatively correlated with the %RH. The kDNA prevalence varied over the months and was negatively correlated with the temperature. Total sandfly abundance did not differ between the sampled habitats, but *P*. *pedifer* was the distinct predominant species only in caves. Moreover, significantly more infected sandflies were found in caves. Only 1/192 rodents were kDNA positive, while 20.0% (5/25) of *Heterohyrax brucei* were found infected.

**Conclusions:**

This study suggests that caves may be a source of multiplication of the infection. If an outdoor control program would be considered, it would be useful to focus on caves in the wet season, when the sandfly abundance is lowest. The captured rodent species appear not important for transmission and the contribution of hyraxes in transmission should be further investigated.

## Author summary

Zoonotic cutaneous leishmaniasis poses a considerable health problem in Ethiopia. Efficient disease control can only be accomplished when all players of transmission are well understood and taken into account. The aim of our study was to investigate in a village in the south, called Ochollo, whether rodents also harbor the parasite and to assess the different potential vector species (sandflies) and their spatial and seasonal distribution. We established that the rodent species we captured are probably no hosts for *Leishmania aethiopica*, but confirmed that hyraxes are abundant and that a high percentage of them is positive for *L*. *aethiopica*. Based on a very large sample size, we found that *Phlebotomus pedifer* is the only vector in the area. We discovered that the general sandfly and specific *P*. *pedifer* abundance are lowest in the wet season and negatively correlated with humidity. We also demonstrated that the sandfly abundance is equally distributed among different habitats, but *P*. *pedifer* and infected sandflies mainly reside in caves. Altogether, we suggest that if outdoor sandfly control methods would be considered, it would be useful to carry it out in the wet season in or around caves. The role of hyraxes in disease transmission should be further investigated.

## Introduction

Cutaneous leishmaniasis (CL) is a vector-borne disease caused by protozoa of the genus *Leishmania* and is listed as one of the neglected tropical diseases (NTD) [1]. It is characterized by nodules or ulcerative skin lesions, which can lead to secondary infections and disfiguring scars. In the Old World, female sandflies of some species of the genus *Phlebotomus* are the vector for *Leishmania*, as they can transmit the parasites when they take a blood meal [2,3]. Since the parasite’s host range includes other mammals aside from humans in Ethiopia, CL is a zoonosis [4].

The annual CL incidence in Ethiopia is estimated around 20 000 to 50 000 cases, which is probably an underestimation [5]. Endemic foci of CL are located in the (mid-)highlands [6], widespread in the country [7,8]. The main causative agent for CL is *L*. *aethiopica* [9] and occasionally *L*. *major* [10] and *L*. *tropica* [11]. The most common vectors are *P*. *pedifer and P*. *longipes* [8,12], although one study also obtained *L*. *aethiopica* promastigotes from *P*. *sergenti* [13].

A steep slope (>2.15 degrees), an altitude between 1700m and 3500m and an average yearly rainfall between 1300 and 1700 mm were found positively correlated with the occurrence of CL [14]. In general, areas with CL are characterized by a temperate climate and rugged environments with cliffs, serving as a favorable habitat for sandflies and hyraxes [15].

Rock hyrax (*Procavia spp*.) and bush hyrax (*Heterohyrax spp*.) are thought to be the reservoir hosts for *L*. *tropica* and *L*. *aethiopica* in Ethiopia [8,12,15], Kenya [16–18] and other African countries [19–22]. In northern Africa, rodents are known to be potential reservoirs for *L*. *major* [23–25] and *L*. *tropica* [26]. Likewise, Ethiopian rodents and bats have recently been found positive for *L*. *tropica* DNA [27,28]. This suggests that rodents and other small mammals, whose burrows can be used by sandflies as resting and breeding sites, might be alternative animal hosts for *L*. *aethiopica*. Resting sites for phlebotomine sandflies can be almost anything, as long as it is relatively cool and humid [29]. The places identified so far are cracks in basalt cliffs, fissures and holes in walls, barns, caves used by hyraxes, rodent burrows, termite nests, soil cracks, tree trunks, etc. [12,29,30].

Ochollo is a village in the mid-highlands of southern Ethiopia where CL is mainly seen among children [7,31]. A study on primary school children in 2014 showed that 4% of the study population had active lesions, 59.8% of them had scars and 1.5% had both, making it a considerable public health problem. Scars and lesions were predominantly localized above the neck with the highest occurrence on the cheeks [7]. One study performed a species typing on skin scrapings of 35 CL patients, confirming the hypothesis that *L*. *aethiopica* causes CL [32].

Former studies described two *Phlebotomus* species in Ochollo: *P*. *pedifer* and *P*. *ashfordi*. *P*. *pedifer* is the most abundant species, shows anthropophilic behavior and is so far the only incriminated vector, though, this information was based so far on relatively small sample sizes [12,33,34]. The parasite species from five naturally infected sandflies was determined and turned out to be *L*. *aethiopica* [33]. During a study in 1973 in Ochollo, *P*. *pedifer* was found to live in close association with hyraxes in cracks in rocks and sandfly blood meals were found to come from humans and hyraxes [9].

This suggested that hyraxes, which are numerous in Ochollo, might play an important role in transmission of *Leishmania* parasites. Ashford *et al*. found four out of 19 *Heterohyrax brucei* (21%) naturally infected with *Leishmania* parasites, and thereby hypothesized that hyraxes might even be able to sustain the parasite in the hyrax population, without a human factor [12].

In this study, we evaluate whether the situation of sandflies and hyraxes is still similar to 45 years ago, we investigate whether also rodents are likely as natural hosts for *Leishmania aethiopica* in the region and we describe spatial and temporal variation in the abundance of (infected) sandflies. Since CL is a zoonosis and the ecology and transmission dynamics are unique to each area, such knowledge must be obtained to lay the groundwork for adequate disease control.

## Methods

### Ethics statement

Animal trapping and sample collection were conducted with authorization of the appropriate institutional and household authorities. Handling of the animals was carried out according to the 2016 Guidelines of the American Society of Mammalogists for use of small mammals in research and education. Permission from EWCA (Ethiopian Wildlife Conservation Authority) was not required according to the Ethical Clearance Committee of Arba Minch University, since our study site was a village which is not part of a protected area and the captured species are not endangered.

### Description of the study area

Ochollo is a village situated approximately 20 km north of Arba Minch at 6°11’N, 37°41’E. It lies on the western side of the Ethiopian Rift Valley, at an altitude of approximately 2100 m. It has a temperate climate, with rainy seasons in February and March (average rainfall 400 mm), and between June and September (average rainfall 600 mm) [31]. The population consists of approximately 5000 people, living on top of hills and across steep slopes. The hills give rise to caves and crevices and the landscape is overall rocky and relatively densely vegetated.

**Fig 1:**
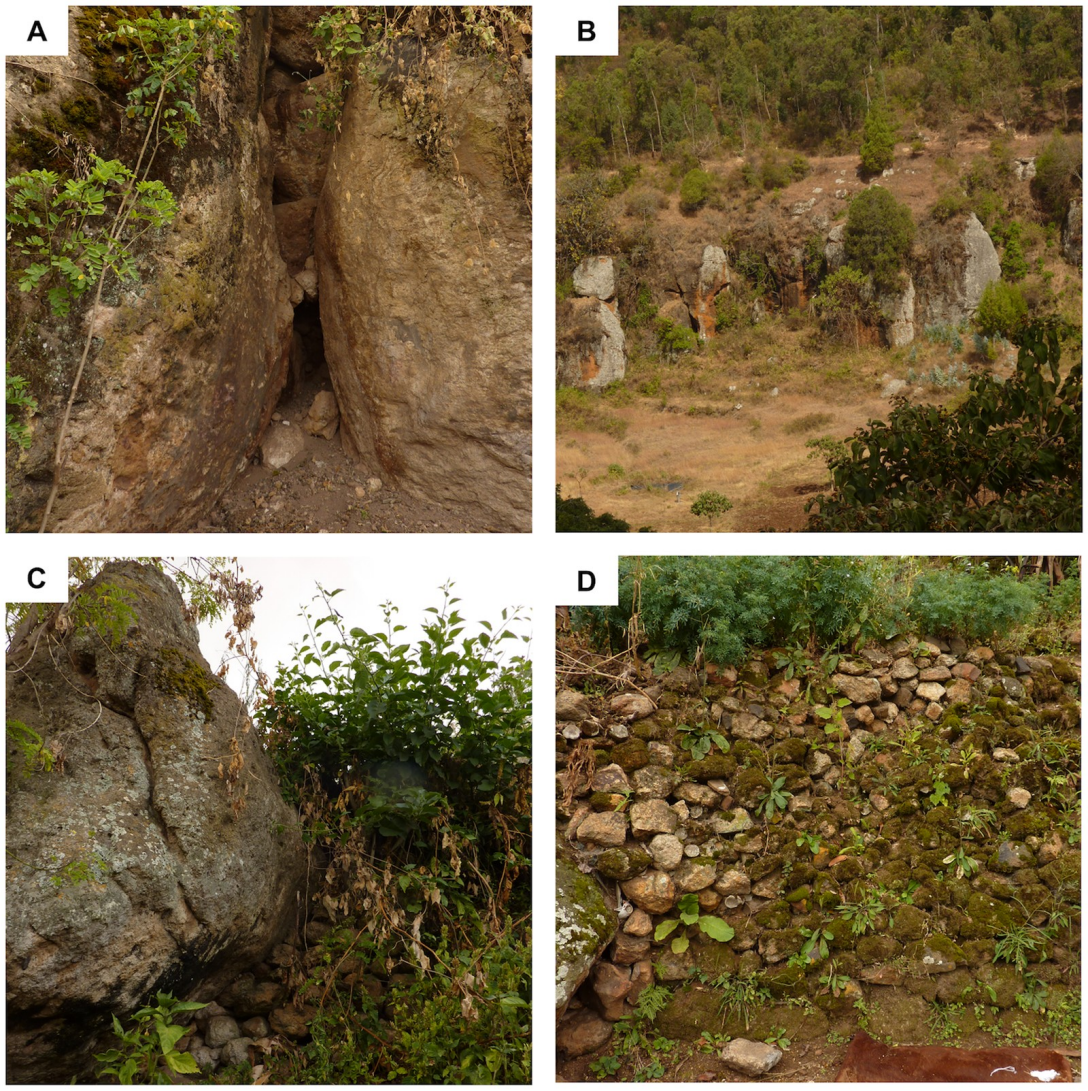
The different sample sites in Ochollo. Three habitats were used for sampling sandflies: (A-B) Caves, which are located on cliffs; (C) Rocky areas, constructed by big boulders; (D) Stone fences, manmade walls by stones covered with moss.

Sampling was carried out in three different habitats: caves, rocky areas and stone fences. Caves (Fig 1A) are highly abundant in Ochollo and are situated in the proximity of houses. They are located on cliffs (Fig 1B), where hyraxes are abundant. Rocky areas (Fig 1C) are a cluster of large boulders, with dark and humid crevices in between, where hyraxes are seen at daytime. Stone fences (Fig 1D) are manmade walls around household compounds and footpaths, constructed by stones from the surrounding area. The stones are covered with moss, indicating a high humidity. There are many small openings between the rocks, leaving space for potential resting sites of rodents and sandflies.

### Sample collection

Sandflies were collected from March 2017 until February 2018, for three consecutive nights between dusk and dawn in the three habitats every month (S1 Table). Sandflies were collected using CDC miniature light traps (John W. Hock Company, USA) and two types of sticky traps covered with sesame oil; sticky traps made from an A4 format cardboard plate covered with white papers and plastic were placed straight at the entrance of crevices, while white laminated A4 format papers were used to fold into cavities (S1 Fig). The traps were equally distributed over the sample sites belonging to the three habitats: caves, rocky areas and stone fences. Female sandflies were sorted out under the microscope. The head, wings and legs were disposed, and the thorax and abdomen were stored in 97% ethanol at -20°C until further analysis.

Rodents were trapped with Sherman live traps (76 x 89 x 229 mm, Sherman Live Trap Co., Tallahassee, FL, USA); in March, April, May, August and September 2017, 400 traps were distributed in three different habitats during three consecutive days, baited with peanut butter and flour and checked daily at sunrise and just before sunset. Hyraxes were collected by local hunters using traditional snare traps.

Mammals were initially identified based on taxonomic features, weighed and measured [35]. Venous blood was collected on filter paper (dry blood spots) and stored at -20°C. Ear samples were collected (4mm x 4mm) and preserved in 97% ethanol at -20°C.

I-buttons (HQMatics, the Netherlands), small climate loggers, were placed at the sampling places to record the temperature and % relative humidity (%RH) on an hourly basis during the whole year. A short description of the weather conditions was recorded on the sampling days.

### Molecular analyses

#### DNA extraction

DNA from individual sandflies was extracted individually by overnight incubation of the thorax and abdomen at 37°C in 50μl extraction buffer (10 mM TrisHCl pH 8, 10 mM EDTA, 0.1% SDS, 150 mM NaCl) and 0.5 μl proteinase K (200μg/ml). Afterwards, 25 μl distilled water was added and the sample was heated for 5 minutes at 95°C [36]. Negative and positive extraction controls were included, respectively being an uninfected and *L*. *major* (MHOM/SA/85/JISH118) infected *Lutzomyia longipalpis* acquired from the ‘Laboratory of Microbiology, Parasitology and Hygiene’ (LMPH, Antwerp, Belgium).

The individual sandfly extracts were cross-pooled in duplicate with an in-house pooling technique per six samples (S2 Fig). For purification of the pools, 1/10th volume 3M NaOAc (pH 5.6) and 2 volumes -20°C 100% ethanol were added to each pool and incubated overnight at -20°C. The samples were centrifuged 15 minutes at 15000 RCF at 4°C and the supernatant was removed. 500 μl of 70% ethanol (chilled at -20°C) was added for washing and the mixture was centrifuged again. After discarding the supernatant, the pellet was left to dry in a heating block at 50°C. Finally, the DNA was resuspended in 10 μl distilled water and left to rehydrate prior to using it for PCR.

DNA from ear and blood samples from rodents and hyraxes was extracted using the NucleoSpin Tissue kit (Macherey Nagel, Germany) according to the manufacturer protocol. Elution was done in 50 μl RNA/DNA free water.

#### Detection of Leishmania DNA

To screen the samples for *Leishmania* parasites, a real-time PCR approach based on Nicolas et al. (2002), targeting 120 bp of the kinetoplast DNA (kDNA) minicircles, was used with minor adjustments [37]. For each reaction, the positive and negative extraction controls described above, as well as PCR controls (RNA/DNA free water and *L*. *major* sandfly extracts respectively), acquired from LMPH, were included. The mastermix was made in a volume of 10 μl, containing 0.8 mg/ml BSA (GE Healthcare Lifescience, Belgium), 1X QUANTIMIX HotSplit Easy kit (Biotools, Spain), 0.1 μM of both primers, JW11 5’-CCT ATT TTA CAC CAA CCC CCA GT-3’ and JW12 5’-GGG TAG GGG CGT TCT GCG AAA-3’ (Invitrogen, Life Technologies, Belgium), and 1 μl template. The reactions were performed on a StepOnePlus Real-Time PCR System (Applied Biosystems, Life Technologies, Belgium). After the initial denaturation for 15 minutes at 95°C, amplification was performed in 40 cycles with ten seconds denaturation at 95°C, ten seconds primer annealing at 58°C and 30 seconds extension at 72°C. For the melt curve, the following program was used: 15 seconds at 95°C, one minute at 60°C followed by a gradual increase of 0.3°C/s to 95°C. Validity of the PCR was determined by the positive control in duplicate that should give the same Ct value (+/- 0.5). The cut-off for positivity was determined by a Ct value of at least 5 Ct values below the lowest Ct value of all negative controls and a melting temperature of approximately 83.8°C (+/- 0.5°C).

#### Leishmania species determination

To identify the *Leishmania* species of the kDNA positive samples, a conventional PCR approach targeting the 350 bp ITS-1 gene based on El Tai et al. (2000) was used [38]. Positive and negative controls were included as described above. The mastermix was prepared in a volume of 15 μl, containing 0.5 μM of both primers, LITSR 5’-CTG GAT CAT TTT CCG ATG-3’ and L5.8S 5’-TGA TAC CAC TTA TCG CAC TT-3’ (Invitrogen, Life Technologies, Belgium), 0.2 mM dNTP (GE Healthcare Lifescience, Belgium), 1X QIAGEN PCR Buffer (Qiagen, Belgium), 0.04 U/μl HotStarTaq DNA polymerase (Qiagen, Belgium) and 1.5 μl template. The PCR was performed on Biometra T professional gradient Thermocycler (Biometra, the Netherlands). After 15 minutes initial denaturation at 95°C, the amplification was performed in 40 cycles of 30 seconds at 95°C, 30 seconds at 53°C and one minute at 72°C, followed by a final extension step of five minutes at 72°C. The results were visualized on a 1.5% agarose gel. Positive samples were sent to ‘Vlaams Instituut voor Biotechnologie’ (VIB, Antwerp, Belgium) for sequencing. Obtained sequences were aligned in GenBank using Basic Local Alignment Search Tool (BLAST) to determine the *Leishmania* species. Sequences showing a query coverage and identity of more than 95% were considered as a successful identification.

#### Sandfly species determination

Given the huge amount of sandflies collected (see further), it was not possible to identify them all morphologically to species level (also because of the effect of the oil on the sticky traps on the body condition), nor to apply molecular identification for all of them. Instead, 200 sandflies were selected from the wettest month (July 2017) and again 200 from the driest month (January 2018) for molecular species determination. Those 400 samples were randomly selected from the trap sites, taking into account the proportion that sandflies from each trap site and habitat contributed to the total amount of sandflies in that month (S2 Table). From the sandflies captured in July 2017, the 200 samples selected for molecular screening included 118 samples from caves, 30 from rocky areas and 52 from stone fences. For January 2018, 124 sandflies were selected from caves, 26 from rocky areas and 50 from stone fences. Furthermore, we also used molecular species identification for all specimens in which kDNA was detected.

For this molecular identification, a conventional PCR targeting the 700 bp cytochrome c oxidase subunit I (COI) based on Kumar et al. 2012 was used [39]. The mastermix was made in a volume of 15 μl, consisting of 0.4 μM of each primer LCO 1490 5’-GGT CAA ATC ATA AAG ATA TTG G-3’ and 0.4 μM HCO 2198 5’-TAA ACT TCA GGG TGA CCA AAA AAT CA-3’ (Invitrogen, Life Technologies, Belgium), 0.2 mM dNTP (GE Healthcare Lifescience, Belgium), 1X QIAGEN PCR Buffer (Qiagen, Belgium) and HotStarTaq DNA polymerase (Qiagen, Belgium). The PCR was performed on a Biometra T professional gradient Thermocycler (Biometra, the Netherlands). After an initial denaturation step of 15 minutes at 95°C, 35 amplification cycles were set at 40 seconds at 94°C, one minute at 72°C and finally seven minutes at 72°C. Since no sequences for *P*. *pedifer* could be found in GenBank, this sandfly species was first morphologically identified and the according COI sequence was obtained with the protocol described above.

#### Small mammal species determination

The species of all captured small mammals was confirmed with a PCR targeting cytochrome B (350bp). The 15μl mastermix contained 1.5 μl template, 2.5 mM MgCl_2_ (Promega, The Netherlands), 0.2 mM dNTP, 0.2 μM of each primer H15915 5’-TCT CCA TTT CTG GTT TAC AAG AC-3’ and L14723 5’-ACC AAT GAC ATG AAA AAT CAT CGT T-3’, 1X GoTaq flexi PCR buffer (Promega, The Netherlands) and 0.03 U/μl GoTaq G2 flexi DNA polymerase (Promega, The Netherlands). The following program was used for amplification: initial denaturation for 5 minutes at 94°C followed by 40 cycles of 30 seconds at 94°C, 30 seconds at 52°C, 1.5 minute at 72°C, and a final extension step of 10 minutes at 72°C.

Amplicons of both PCRs were loaded on 1.5% gel. Successful samples were sent for sequencing at VIB and sequences were aligned in BLAST for species identification with the same conditions for interpretation as mentioned for ITS-1 sequences.

### Statistical analysis

The statistical analysis was performed with R version 3.4.3. Statistical tests were considered significant when the p-value < 0.05.

#### Differences in sandfly abundance

We tested whether the species distribution, over the different habitats, varied in the wettest and driest month by a generalized linear model (GLM, Poisson) using the glm function in R. The response variable was the number of sandflies of a particular species, in a specific habitat, within one month. The effects were habitat type, month, and species, and the interaction effects between the three factors. The histogram and Shapiro Wilk test on the residuals of the model indicated a normal distribution. Significance testing was done using an ANOVA Chi-square test on the model.

We tested whether the total sandfly abundance varied between habitats and seasons. We fitted general linear mixed models (GLMM) using the R package lmerTest [40]. The response variables for this model were the logarithmically transformed mean numbers of females or males, within one of the three habitat types, for a specific sample site, within one month. The fixed effects were sex, month and habitat type and interaction effects between the three fixed effects were included. Additionally, the random effect sample site nested within habitat type (different sample sites were used for each habitat type, all assigned with a unique code) was added to the model. Both, the histogram and Shapiro Wilk test on the residuals of this model indicated a normal distribution. Tests of fixed effects were based on F-tests with degrees of freedom approximated using Satterthwaite’s method.

The correlation between the mean monthly temperature and %RH and the total sandfly abundance was determined. Two separate mixed models were constructed with either mean temperature or % RH as explanatory variable, because the variables were strongly correlated. The models were constructed with the fixed effects mean monthly temperature or %RH, habitat and their interaction. The random effect, response variables and the procedure of significance testing were the same as described above.

#### Differences in infection rate

Prevalence was always calculated based on the number of tested female sandflies. A general linear model (GLM) was coded with R package lme4 [41] to evaluate whether the kDNA positive sandflies are more abundant in certain habitats and show seasonal fluctuations. The response variables for this model were the proportions of kDNA positive sandflies per sample site per month. We added a very small number (0.005) to each of the proportions to avoid numerical problems in categories with overall zero proportions. The fixed effects were month, habitat type and their interaction. The optional vector ‘weights’ was included in the model, to indicate that the proportion was calculated based on the tested sandflies. The ANOVA Chi-square test on this model indicated which parameters had a significant effect. Additionally, a general linear hypothesis test (packages multcomp and zoo [42,43]) was used as a multiple comparison function, specified as a Tukey test, for the habitat type.

Besides, a GLM was generated to assess whether the temperature and %RH were correlated with the proportion of kDNA positive sandflies. The same steps were undertaken as described before for two separate models, with the mean monthly temperature or %RH, habitat type and the interaction as parameters, and the proportion of kDNA positive sandflies per sample site per month as response variable. The Chi-square test indicated which parameters had a significant effect.

## Results

### Small mammals, parasites and vectors

Small mammals were captured and subsequently screened with different PCR approaches (Table 1). We caught 192 rodents, of which one ear sample, derived from *Mus mahomet*, was positive for kDNA. The sample was not positive for ITS-1, thus the *Leishmania* species could not be determined. Of the three shrews (*Crocidura olivieri*) and four bats (*Nycteris spp*.) that were captured, none were found infected. From 25 captured hyraxes, which were all *H*. *brucei*, five (20.0%) ear samples gave a positive result for kDNA. Of these five samples, four were positive for ITS-1 and when aligned in GenBank, all were *L*. *aethiopica*. Lesions were never seen on the animals’ nose or ears. None of the dry blood spot samples was positive.

**Table 1:** Small mammals captured in Ochollo and the amount (proportion %) of kDNA and ITS-1 positives.

Small mammal species	# captured	kDNA+ (%)	ITS-1+	BLAST
**Rodents**				
*Arvicanthis spp*.	24	0		
*Dendromus spp*.	3	0		
*Lophuromys cf*. *chrysopus*	24	0		
*Mus cf*. *proconodon*	21	0		
*Mus mahomet*	3	1 (33.3%)	0	
*Mus minutoides*	1	0		
*Rattus rattus*	41	0		
*Stenocephalemys albipes*	75	0		
Total rodents	192	1 (0.5%)		
**Other small mammals**				
*Crocidura olivieri*	7	0		
*Nycteris spp*.	4	0		
*Heterohyrax brucei*	25	5 (20.0%)	4	*Leishmania aethiopica*

An overview of the sandfly data is presented in S1 Table. In total, 17190 (*Sergentomyia* and *Phlebotomus*) sandflies, 8410 females (48.9%) and 8780 males (51.1%), were caught between March 2017 and February 2018. Of the selection of 400 specimens from different habitats collected in January and July, 293 (73.3%) were *P*. *pedifer* (Table 2), the others were mostly *Sergentomyia spp*. and a very small number of *Phlebotomus* that matched with < 95% identity in GenBank with different *Phlebotomus* species. The overall species composition did not differ between months ($\chi_{6}^{2}=1.03$, p = 0.598).

**Table 2:** Proportions of sandfly species in different habitats during the wettest (July 2017) and driest (January 2018) months in Ochollo.

Month	Species	Cave	Rocky area	Stone fence	All habitats
July 2017	*Phlebotomus pedifer*	93 (78.8%)	16 (53.3%)	32 (61.5%)	141 (70.5%)
	*Sergentomyia spp*.	22 (18.6%)	13 (43.3%)	19 (36.5%)	54 (27.0%)
	Other *Phlebotomus*	3 (2.5%)	1 (3.3%)	1 (1.9%)	5 (2.5%)
January 2018	*Phlebotomus pedifer*	117 (94.4%)	19 (76.0%)	16 (31.4%)	152 (76.0%)
	*Sergentomyia spp*.	7 (5.6%)	5 (20.0%)	35 (68.6%)	47 (23.5%)
	Other *Phlebotomus*	0 (0.0%)	1 (4.0%)	0 (0.0%)	1 (0.5%)
Overall seasons	*Phlebotomus pedifer*	210 (86.8%)	35 (63.6%)	48 (46.6%)	293 (73.3%)
	*Sergentomyia spp*.	29 (12.0%)	18 (32.7%)	54 (52.4%)	101 (25.3%)
	Other *Phlebotomus*	3 (1.2%)	2 (3.7%)	1 (1.0%)	6 (1.5%)

Of 8410 females, 1065 were excluded for parasite screening, because of contamination or inhibition prior to or during DNA extraction (S1 Table). The reactions were determined as invalid, because positive and negative extraction controls did not give correct results. The overall percentage of sandflies positive for kDNA was 2.6% (187/7345). Assuming an overall proportion of 73.3% *P*. *pedifer* in the total sandfly population (Table 2), the proportion of the kDNA positive *P*. *pedifer* is 3.5% (187/5384). Of the 187 kDNA positive samples, 162 were positive for ITS-1 (86.6%, S3 Fig), of which 58 samples were successfully sequenced (35.8%). The established species was *L*. *aethiopica* in all cases. The COI sequence of some morphologically identified *P*. *pedifer* sandflies was used as a reference sequence to align the obtained sequences with. Of the 187 kDNA positive samples, 148 were positive for COI (79.1%, S4 Fig), of which 147 samples were successfully sequenced (99.3%). All of the kDNA positive sandflies were identified as *P*. *pedifer*.

### Seasonal changes of the sandfly abundance and infection rate

**Fig 2:**
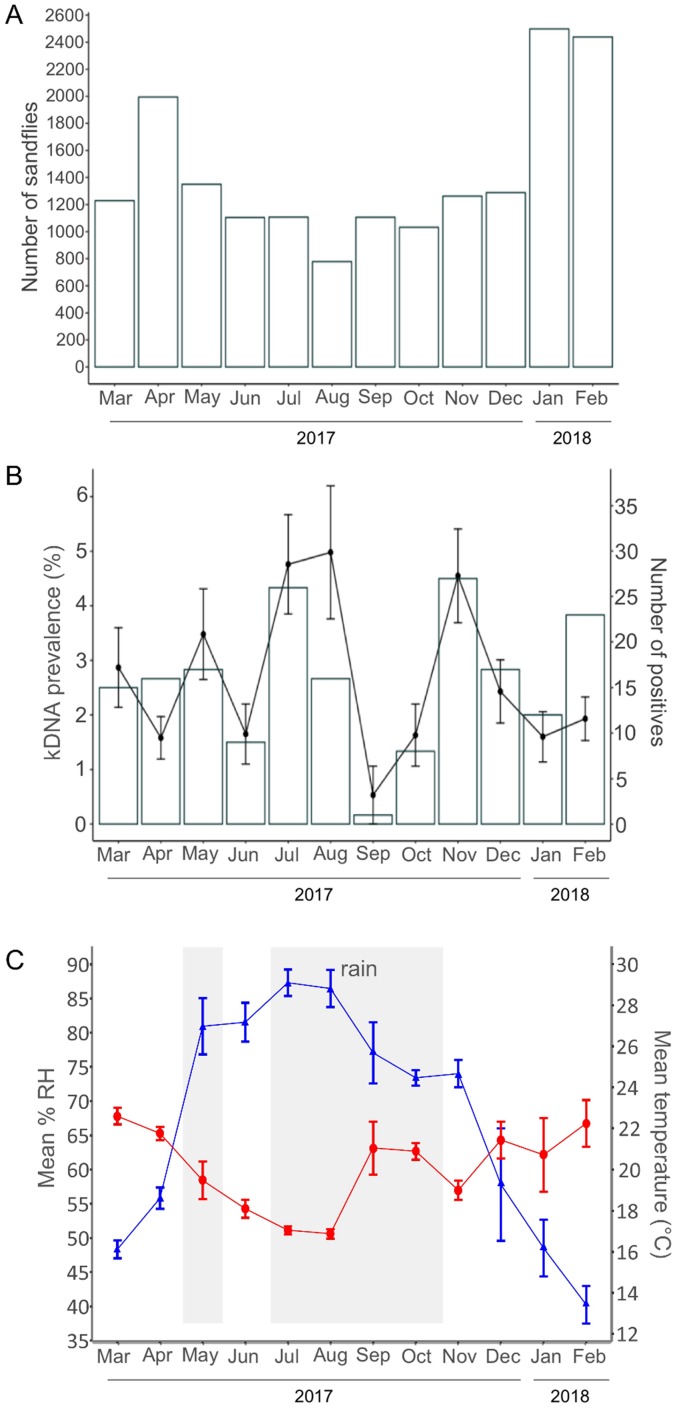
Monthly fluctuations of the (infected) sandflies and seasonal parameters (March 2017—February 2018). (A) total number of sandflies caught per month. (B) the mean kDNA prevalence (%) among tested female sandflies per month with standard errors on the left axis (line plot) and the number of positive sandflies per month on the right axis (bar plot). (C) The mean monthly % RH (left axis) and temperature (°C, right axis) and according standard errors are respectively shown by a blue triangle and red circle line. The months when there was rainfall during the time of sandfly collection are depicted with grey background.

An overview was made for the overall monthly number of captured sandflies and proportion of kDNA positive females (Fig 2A and 2B). Fluctuations in the abundance of sandflies ranged between 779 and 2498 sandflies per month and was significantly different over the months (F_11,160_ = 6.99, p < 0.001). The sandfly abundance was higher in the recorded dry season, with peaks in January, February and April. The population was lower from June to October, with the lowest point in August, which corresponds with the documented rainy season. Since we found no difference in the proportion of *P*. *pedifer* between the extreme months (Table 2), we assume that the seasonal patterns for the total sandfly population can be taken as a proxy for those in *P*. *pedifer* alone.

The kDNA prevalence among female sandflies varied significantly over the months ($\chi_{11}^{2}=29.73$, p = 0.002), fluctuating from 0.5% to 5.0%, however the Tukey test could not show pairwise differences between months. There were peaks in kDNA prevalence rates in July and August and November. Similarly, the number of infected sandflies was highest in July and November, but also in February a relatively high number of infected sandflies was obtained, while the prevalence rate was rather low. A steep decline was observed in September, in both absolute numbers and prevalence. However, only 34% of the specimens from this month were successfully tested.

### Correlation between seasonal parameters and sandfly abundance/infection rate

The monthly temperature and % RH were recorded in Ochollo from March 2017 to February 2018 (Fig 2C and S1 Table). Average values were used, since the recordings of different habitats were indistinguishable. The mean monthly temperature ranged between 17.0°C and 22.6°C over the year, while the % RH stretched between 40.2% and 87.3%. Mean temperature and % RH had an inverse pattern. The mean % RH rose from March to August and dropped from August to February. It showed a steep decline from November to February and had its lowest value in February, while the sandfly abundance peaked in January and February (2498 and 2439 sandflies respectively). The temperature fell from March to August and from then on increased again. It dropped to its lowest recordings in July and August (17.1°C and 17.0°C), while the prevalence then peaked highest (4.8% and 5.0%). In September and October, the temperature increased (21.0°C and 21.1°C) and simultaneously the prevalence decreased (0.5% and 1.6%). In November, a slight drop in temperature (about 2°C) was recorded and the number of positive sandflies increased from eight in October to 27 in November. The months that were recorded to have had rainfall during the sandfly collection were May, July, August, September and October.

The correlations between the mean temperature and % RH, and the average monthly number of sandflies and kDNA prevalence were obtained by GLMMs and GLMs respectively. Temperature did not have a correlation with the sandfly abundance (F_1,217_ = 0.37, p = 0.955), but the % RH showed a significant negative correlation (F_1,217_ = 5.95, p = 0.015). There was no interaction effect, meaning that the type of habitat (cave, stone fence or rocky area) did not influence the association between temperature and % RH and the population.

Temperature was negatively correlated with kDNA prevalence ($\chi_{1}^{2}=3.91$, p = 0.048), while the % RH showed a borderline non-significant positive correlation with the kDNA prevalence ($\chi_{1}^{2}=3.79$, p = 0.051). The type of habitat did not influence the association between temperature and the kDNA prevalence, but there was an interaction effect between the type of habitat and the mean % RH ($\chi_{2}^{2}=6.87$, p = 0.032).

### Spatial (monthly) abundance of the (infected) sandflies

**Fig 3:**
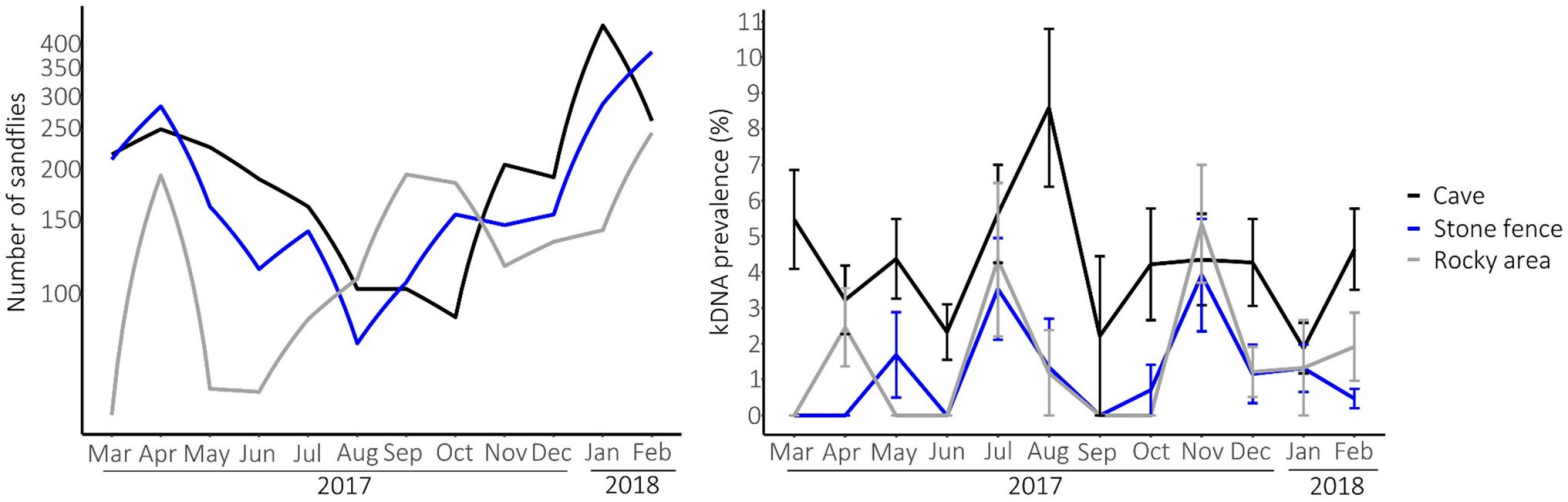
The average monthly sandfly abundance and kDNA prevalence (%) at the three sampled habitats (cave, stone fence, rocky area) in Ochollo (March 2017—February 2018). Left panel: the average number of sandflies caught per month at each habitat presented on a logarithmic scale. Right panel: monthly kDNA prevalence (%) among tested females for each habitat. Error bars were calculated based on the standard error and mean, but were too small to visualize on the left graph. Black, blue and grey lines respectively represent the sandflies and kDNA prevalence at caves, stone fences and rocky areas.

The average monthly sandfly abundance at each habitat type is presented on a logarithmic scale (Fig 3, left panel). Sandflies were present in each habitat during the whole year. The monthly variation of the sandfly abundance was significantly different at each habitat type (F_22,160_ = 2.40, p < 0.001), as shown by the crossing lines and different peaks and drops for each habitat type, indicating that the sandfly population was not predominantly present at one of the three habitats. From a total number of 400 sandflies, 200 from July 2017 and 200 from January 2018, the number and proportion of each sandfly species in the different habitats are presented in Table 2. The three-way interaction of the model on the selection of 400 sandflies of which the species was determined, indicated that the abundance of sandflies in the different habitats was significantly different in July 2017 and January 2018 ($\chi_{0}^{2}=23.62$, p < 0.001). Only in caves, the population mainly consisted of *P*. *pedifer* in July (93/118, 78.8%) and January (117/124, 94.4%), while raw numbers and proportions of *P*. *pedifer* were lower at rocky areas and stone fences.

The monthly kDNA prevalence among tested females (Fig 3, right panel) followed a similar pattern at each habitat type ($\chi_{22}^{2}=32.04$, p = 0.077) and the habitat had a significant correlation with the prevalence ($\chi_{2}^{2}=55.01$, p < 0.001). Throughout the whole year, infected sandflies were present at caves, in contrast with the stone fences and rocky areas, where there were several months without infected sandflies. At all three habitats, a peak in prevalence occurred in July or August. At stone fences and rocky areas, the maximum prevalence was reached in November.

**Fig 4:**
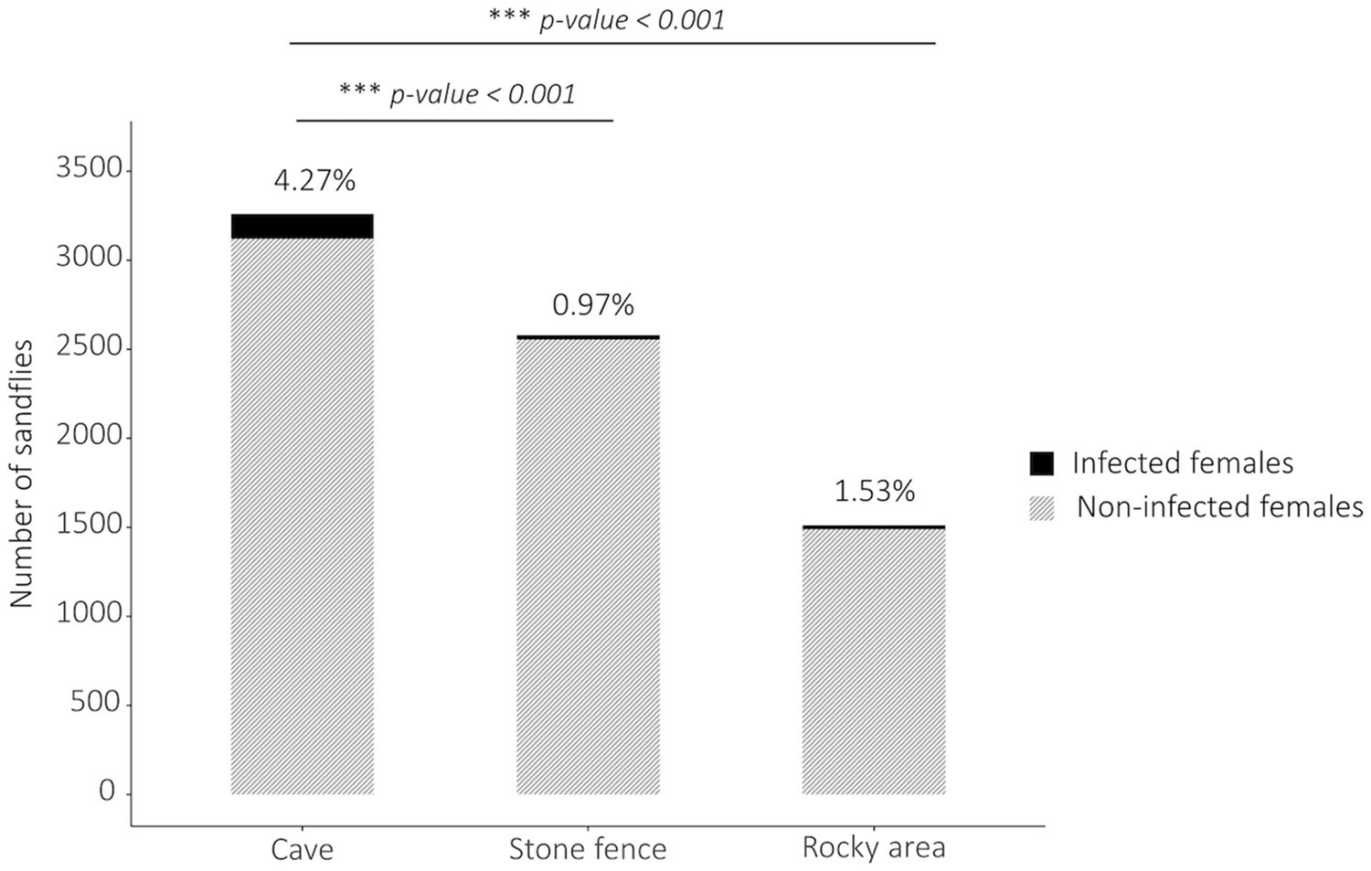
The distribution of (infected) sandflies in different habitats. The total number of non-infected and infected female sandflies in each of the three sampling habitats (cave, stone fence, rocky area) are respectively presented by the striated grey and solid black parts of the bars. P-values were obtained by a Tukey test.

Fig 4 presents which habitat was preferred by (infected) sandflies. According to the Tukey test, caves (4.27%) had a significantly higher prevalence than stone fences (0.97%, p < 0.001) and rocky areas (1.53%, p-value <0.001), which is also reflected by the black curve (Fig 3 left panel) that is, except for November, consistently higher than the other two. Raw numbers are presented at the bottom of S1 Table.

## Discussion

This work presents results on the parasites, small mammals and sandflies that contribute to CL transmission in southern Ethiopia, as well as the seasonal dynamics and habitat distribution of (infected) sandflies.

### Small mammals, parasites and vectors

We demonstrated that a high proportion of *H*. *brucei*, which are abundant in Ochollo, were asymptomatically infected with *L*. *aethiopica*, confirming the results of Ashford et al. and Lemma et al. [8,12]. Similarly, *Procavia spp*. are described to carry *L*. *tropica* parasites in Ethiopia [12,15], Kenya [17] and other African countries [19–22], with prevalence ranging between 3.5% and 27% with seasonal variations. However, since the nomenclature *L*. *aethiopica* was only named as such in 1973, the aetiology of CL in Ethiopia was believed to be *L*. *tropica*, implying that the infections found in Ethiopia were in fact most probably due to *L*. *aethiopica* [9]. The high abundance and infectivity rate of hyraxes in Ochollo, in combination with the fact that they can become up to 13 years old, suggests that hyraxes might play a considerable role in the transmission of zoonotic CL, as suggested already 45 years ago. Yet, it remains to be evaluated how long the parasite can be sustained in a hyrax and how efficient it can be transmitted to *P*. *pedifer* [44]. Our study also assessed whether other small mammals could contribute to the transmission of CL. Only a single *Mus mahomet* was found kDNA positive, indicating that the captured rodent species do not play a major role as a source of infection in southern Ethiopia. Abebe et al. found one ground squirrel (*Xerus rutilus*) naturally infected with *L*. *aethiopica* in Aba Roba (1200 m), a VL endemic area, where there has never been a human case of CL [45]. Except for this report, to our knowledge *L*. *aethiopica* has not been found in rodents [8,12,46,47]. In contrast, studies carried out in different areas in Ethiopia found *L*. *tropica* in *Arvicanthis sp*., *Gerbillus nanus* and *Acomys spp*. [13,28] and *L*. *major* in *Arvicanthis niloticus* [48]. We did not obtain enough samples from bats and shrews to draw any meaningful conclusions from this study. It would be interesting to acquire knowledge about the blood meal sources of *P*. *pedifer* in the area. If particular rodent species or other mammals appear to be a dominant blood source, new work could target these species. All dry blood spots samples were negative for kDNA, suggesting that these samples should not be used for molecular detection of *L*. *aethiopica*.

Based on a selected subsample of 400 sandflies, it can be concluded that *P*. *pedifer* (73.3%) is the predominant sandfly species in Ochollo. The rest of the sandflies mainly belong to species in the subgenus *Sergentomyia*. Only 1.5% of the sandflies in our traps were other *Phlebotomus* species. The exact species could not be determined, because sequences were not available in GenBank, but most probably it was *P*. *ashfordi* [33,34]. Overall, 2.6% of the sandflies (or an estimated 3.5% of *P*. *pedifer*) captured between March 2017 and February 2018 in Ochollo were positive for kDNA. CL has been described as a disease of childhood in Ochollo, as about 65% of primary school children had scars or active lesions [7]. The infection at young age might be due to the children’s or sandflies’ behavior, but it could also be explained by the high prevalence and sandfly abundance, which indicate that there is a high intensity of *Leishmania* transmission, increasing the risk of early exposure to an infectious sandfly. Studies on the behavior of children and indoor/outdoor biting behavior of sandflies should be carried out to find out where and when transmission takes place. Two prior studies carried out in Ochollo described that promastigotes were found in 5.4% (2/37) [12] and 1.67% (5/359) [33] of the dissected sandflies. They only dissected a small number of *P*. *pedifer* females, in contrast with the current research, where all sandflies (*Phlebotomus* and *Sergentomyia*) were tested. The former two studies proved that *P*. *pedifer* is a vector for *Leishmania* by dissecting the midgut of the sandflies, while this study intended to evaluate whether other sandfly species could also carry *Leishmania* parasites. Therefore, we opted to screen a large sample size with a real-time PCR targeting kDNA. *Leishmania* parasites have a concatenated network of kDNA minicircles, which are highly abundant, making it a very sensitive fragment to target [49]. *P*. *pedifer* was the only infected sandfly species in Ochollo carrying exclusively *L*. *aethiopica* parasites. There is very limited knowledge about this vector, although this is a prerequisite for efficient vector control strategies. To know when and where to deal with the vector population, the seasonality and habitat preference were determined.

### Seasonality

The relative humidity seemed to be a good indicator for rainfall, as rainfall on the days of sampling was recorded in most months with a high relative humidity. The seasons did not appear like previously described for this area [31], confirming that seasons are changing, as mentioned by inhabitants.

Our data illustrate that the sandfly abundance and *Leishmania* infection in Ochollo were present throughout the whole year and exhibit a distinct seasonality. Since sandflies were not collected in the same number of caves, stone fences and rocky areas every month, this might give a slightly biased representation of the monthly abundance of sandflies. The % RH was found negatively correlated with the sandfly abundance, and since the % RH is a good proxy for rainfall, there is a decline in the sandfly abundance when the rainfall increases. This was reflected by the sandfly abundance, which peaked during the dry months and dropped in the rainy period. With *P*. *pedifer* accounting for nearly 75% of all sandflies, in the wet as well as in the dry season, it can be stated that the species’ population decreases with increasing rainfall. A study in the Mt. Elgon region in Kenya by Mukhwana et al. observed the same fluctuation in the abundance of *P*. *pedifer*, showing a peak in the two dry seasons and a drop in the two wet seasons [50]. Although that study was only based on 657 sandflies, it supports the current findings. No other studies were carried out on seasonality in areas where *P*. *pedifer* is responsible for CL transmission. *P*. *longipes* is the other main vector for *L*. *aethiopica* in Ethiopia and shows morphological and ecological similarities with *P*. *pedifer* [9,51]. However, contradictory to the obtained results for *P*. *pedifer*, an increase in rainfall was accompanied with a rise in the *P*. *longipes* population in a similar study conducted in Kutaber [12].

Increasing temperatures appeared to be accompanied with a drop in the number and proportion of kDNA positive sandflies, but not as distinct as the correlation between temperature and sandfly abundance. The low prevalence rate in September should be interpreted with caution. Due to inhibition, only 34% of the female sandflies caught in September were successfully tested, which might slightly lower the strength of the prevalence estimation within this month.

Studies have been done to investigate the effect of temperature on the development of *Leishmania* parasites in sandflies [52], as well as the metabolism of the sandflies [53]. Metabolic processes are slower at lower temperatures, so there is a delay in defecation when temperatures are lower, providing more time for *Leishmania* to establish an infection in the midgut [52,53].

Contradictory to our results, the seasonality of *P*. *pedifer* infected with *L*. *aethiopica* in the Mt. Elgon region in Kenya showed two drops, concurrent with the rainy seasons [50]. However, based on the tables and figures provided in the paper, it is unclear how the presented results were calculated: only 21 females were infected with promastigotes, yet four trapping sites over a period of 12 months were described, resulting in 48 separate prevalence values. None of them were zero, raising doubts about the presented results. In Kutaber, the infection of promastigotes in *P*. *longipes* varied considerably over 14 months, but seemed to be independent of seasonal conditions [12].

### Spatial abundance

Insight in the spatial abundance of the vector is important for potential future outdoor vector control strategies. The overall sandfly population in Ochollo was not predominantly abundant at a particular habitat, though, hyrax feces suggested to serve as larval food, were only present at caves and rocky areas. It was remarkable that in caves, on average almost 87% of the sandflies were *P*. *pedifer*, while at stone fences and rocky areas, which are situated closer to the people’s houses, only half or even less than half of the population was *P*. *pedifer*. Other papers describe without statistical analysis that the preferred habitat for *P*. *pedifer and P*. *longipes* are cracks in basalt cliffs (here referred to as caves) [12,33,54,55].

The infected sandfly population was evidently more present at caves, where they were found throughout the whole year, while stone fences and rocky areas had several months without kDNA positive sandflies, implying that caves could be the source of multiplication of the infection. A study in Mt Elgon Region in Kenya observed that CL cases as well as *P*. *pedifer* were mainly found near caves and concluded from this that human infection with *L*. *aethiopica* by *P*. *pedifer* is happening near caves. It must be mentioned though, that no other possible habitats of *P*. *pedifer* were considered in that study [55]. At first sight, there seemed to be more CL cases close to the caves in Ochollo, but more research is required to investigate where the transmission particularly happens.

### Public health relevance

Data on vectors and reservoirs of CL in southern Ethiopia are very limited. Until now, there are no efficient intervention programs, partially due to a potentially important zoonotic component and ecological factors associated with the disease that are left aside. Moreover, CL is moving towards new areas with susceptible people, because people are building settlements and cultivate closer to habitats of hyraxes and sandflies [8]. Our results can provide guidance for disease management programs in areas in southern Ethiopia and Kenya with a similar ecological context. Although there is still need to investigate potential indoor transmission, if outdoor vector control would be considered, it would be a good idea to focus on caves in the beginning of the wet season, when the population is at its minimum. The role of hyraxes in CL transmission should be further investigated to assess whether they should be included in control programs. Destroying hyrax habitats close to human settlements is almost impossible in Ochollo, since the majority of the houses are surrounded by basalt cliffs in a range of 200 m. Shooting hyraxes or biological control of the population is believed not to be effective and might result in an increase in human-vector contact [4,8]. Rather than focusing on the hyraxes, attractive toxic sugar treated barrier fences around caves could be used to prevent sandflies from going towards human dwellings to acquire a blood meal [56]. Also, people should be made aware of the risk they face when building settlements or residing in the proximity of caves [57]. Additional information is required about the biting behavior of *P*. *pedifer* to decide whether indoor residual spraying and insecticide impregnated bed nets could be beneficial.

## Supporting information

S1 Fig Traps used for sandfly collection. A) CDC miniature light trap placed inside a cave; B) sticky traps within a cave; C) laminated papers rolled up into crevices of a stone fence.
(TIF)

S2 Fig Pooling of sandfly extracts before purification. Pooling was carried out vertically (PV = pool vertical) and horizontally (PH = pool horizontal) per 6 samples. Row G and H do not contain any samples. When two pools appear positive (in this case i.e. PH1 and PV3) after screening, the cross-over represents a kDNA positive sandfly.
(TIF)

S3 Fig ITS-1 amplicons on a PCR gel. ITS-1 amplicons show a band around 350bp. A 100bp ladder was used and, on the right, a positive control (band at 350bp) and negative control (no band) are depicted.
(TIFF)

S4 Fig COI amplicons on a PCR gel. COI amplicons show a band around 700bp. A 100bp ladder was used and the positive (700bp band) and negative control (no band) are shown.
(TIFF)

S1 Table Overview of the monthly sample collection in Ochollo (March 2017—February 2018). The total number of sandflies, males and females caught per month. The number of not tested and tested females per month. The percentage of tested females, the number of kinetoplast DNA (kDNA) positive females and the kDNA prevalence (%) per month.
(PDF)

S2 Table Overview of the total collection of sandflies and the selection that was used from each habitat and specific sample site for species determination. For species identification, 200 sandflies were collection from July 2017 and again 200 from January 2018. Sample sites are the different places where sandfly traps were placed, which contribute together to the total collection in a particular habitat. ‘Collection’ describes the number of sandflies from a particular habitat or trap site that contributed to the total amount of sandflies collected during that month. This same proportion (percentages) was used for the sandflies that were brought to species level, here referred to as ‘selection’.
(PDF)

S1 Data Dataset of sandfly captures in Ochollo (March 2017—February 2018) and results of molecular analyses. (CSV)

S2 Data Dataset of the captured rodents and hyraxes in Ochollo (March 2017—February 2018) and results of molecular analyses. (XLSX)

S3 Data Data of the temperature and humidity loggers from different habitats in Ochollo (March 2017—February 2018). (XLSX)

S4 Data Species data of 200 sandflies from the wettest (July 2017) and again 200 from the driest (January 2018) months in Ochollo. (XLSX)
