# Supplementary figures and images for: Ecology and seasonality of sandflies and potential reservoirs of cutaneous leishmaniasis in Ochollo, a hotspot in southern Ethiopia
Source: PLoS Negl Trop Dis. 2019 Aug 19;13(8):e0007667. doi: 10.1371/journal.pntd.0007667 (PMC6715250; doi:10.1371/journal.pntd.0007667)

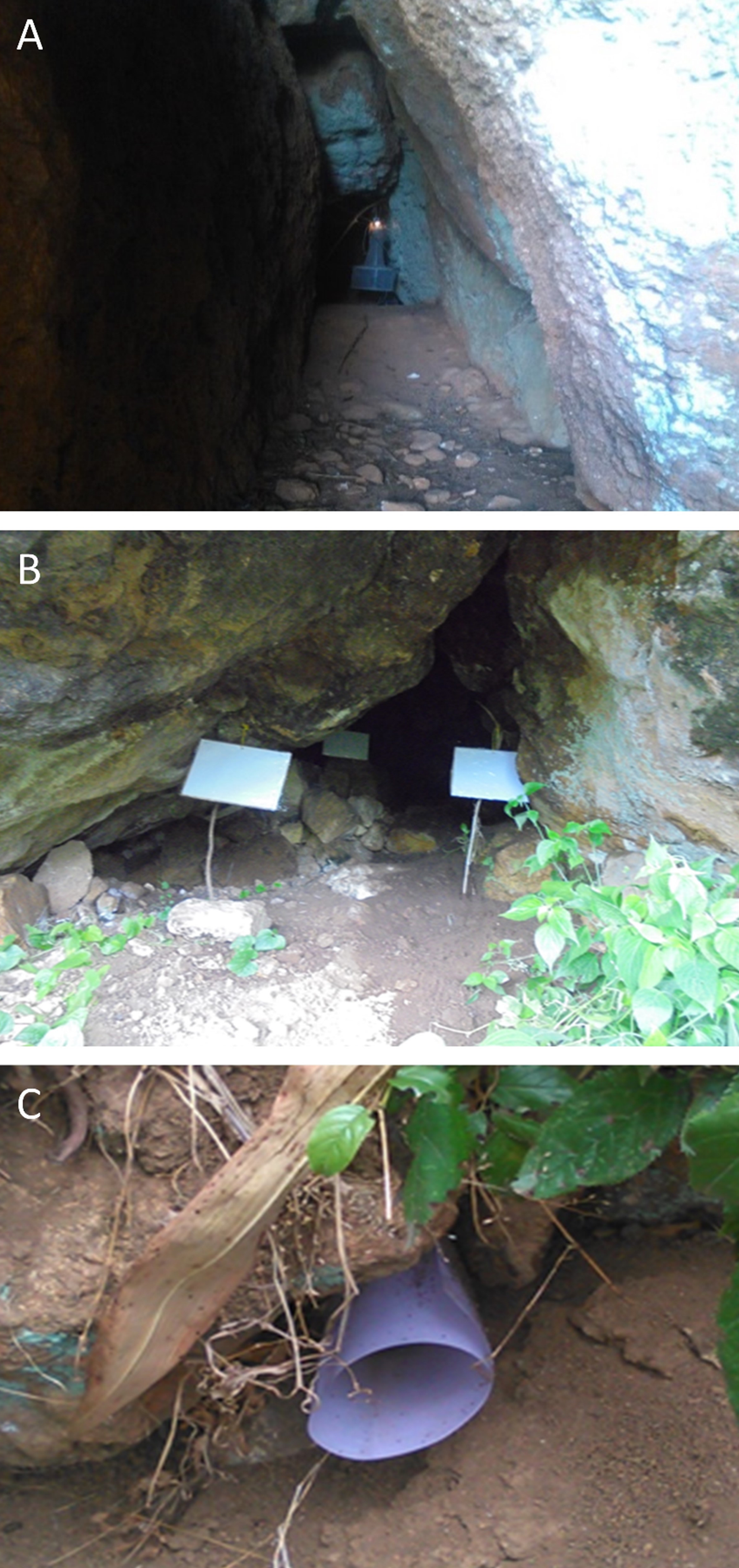

Supplement: S1 Fig — A) CDC miniature light trap placed inside a cave; B) sticky traps within a cave; C) laminated papers rolled up into crevices of a stone fence. (TIF) [file pntd.0007667.s001.tif]

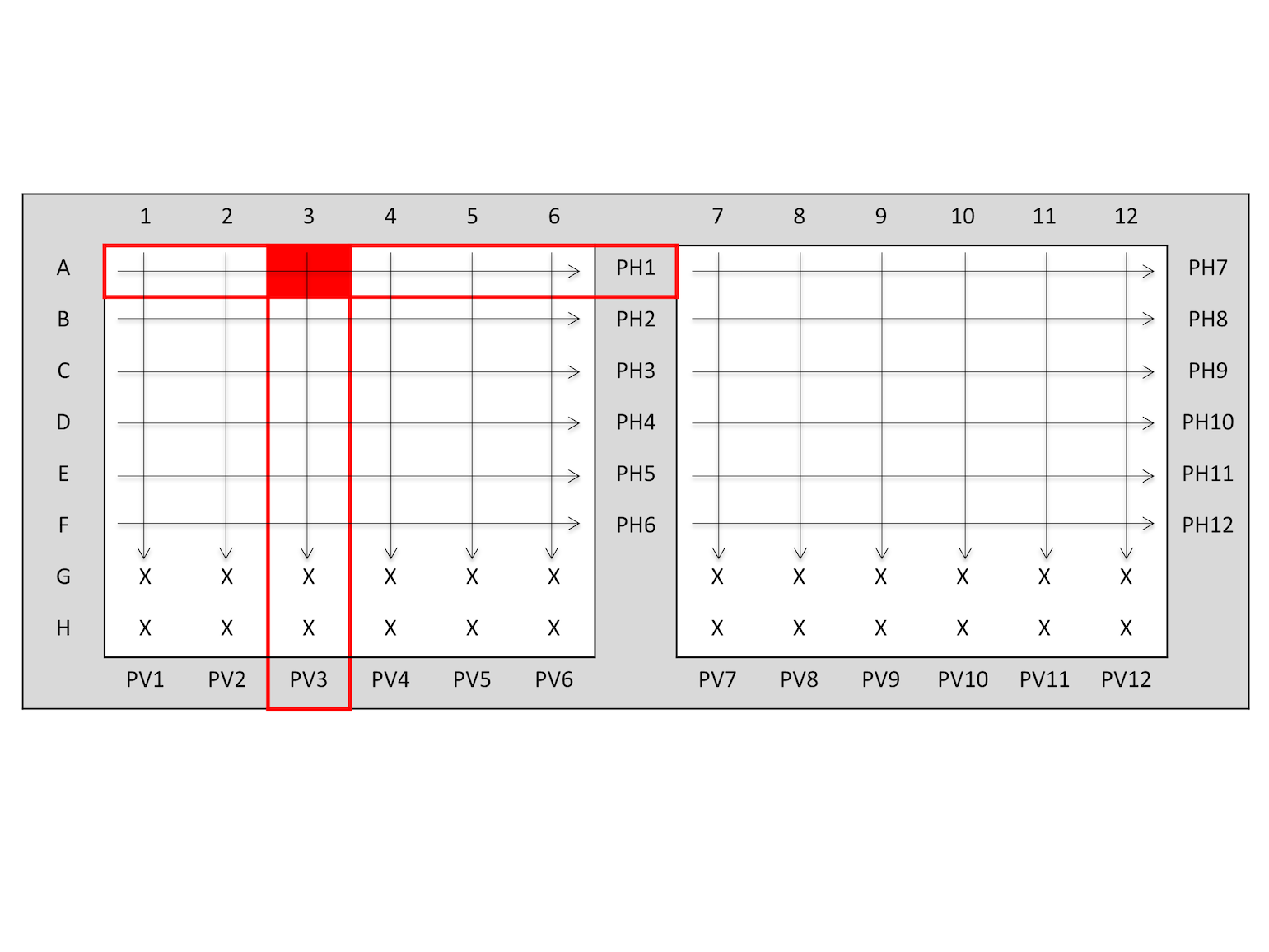

Supplement: S2 Fig — Pooling was carried out vertically (PV = pool vertical) and horizontally (PH = pool horizontal) per 6 samples. Row G and H do not contain any samples. When two pools appear positive (in this case i.e. PH1 and PV3) after screening, the cross-over represents a kDNA positive sandfly. (TIF) [file pntd.0007667.s002.tif]

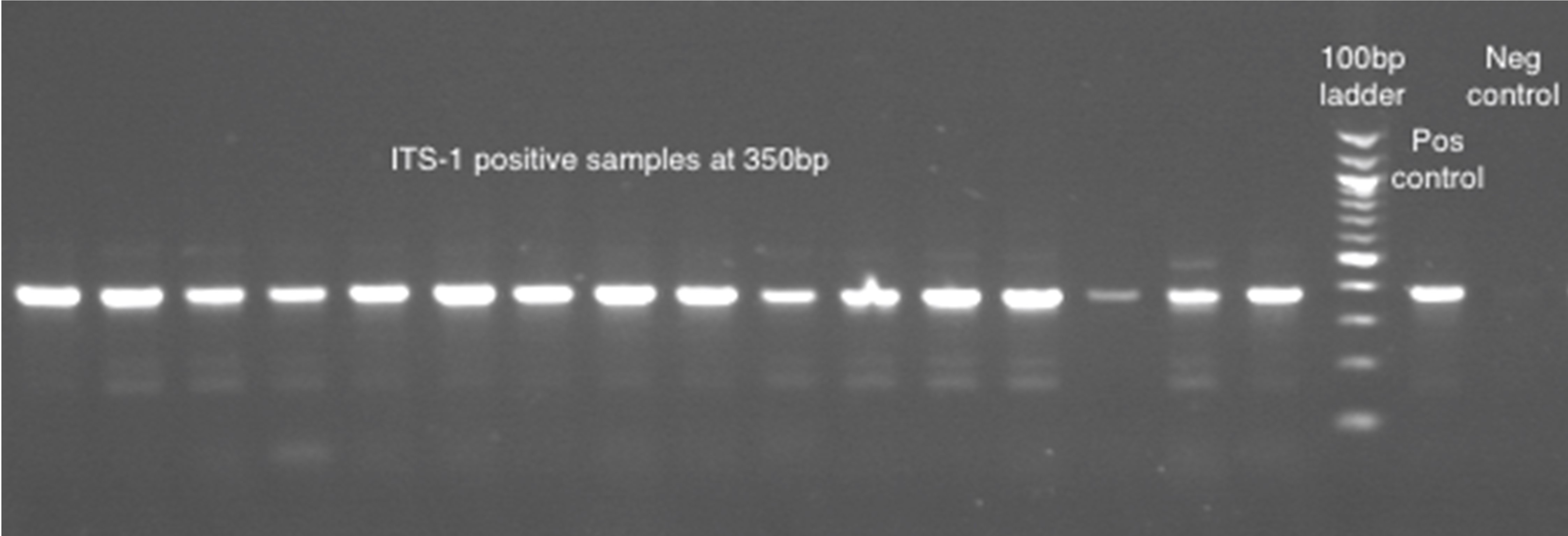

Supplement: S3 Fig — ITS-1 amplicons show a band around 350bp. A 100bp ladder was used and, on the right, a positive control (band at 350bp) and negative control (no band) are depicted. (TIFF) [file pntd.0007667.s003.TIFF]

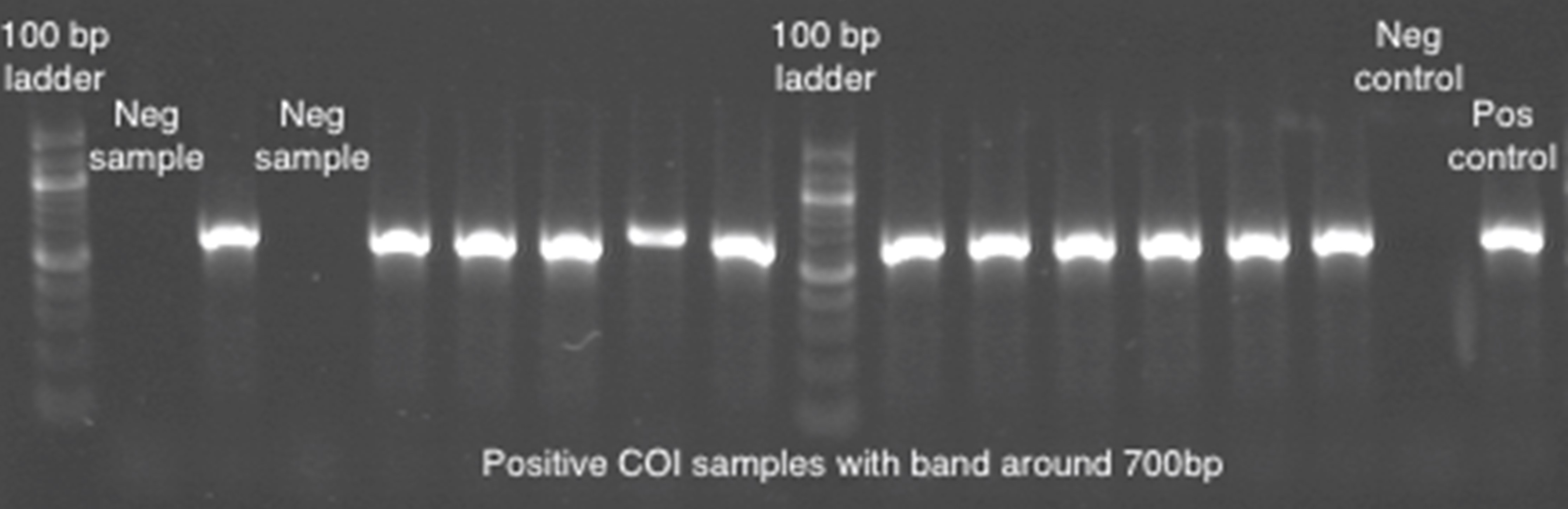

Supplement: S4 Fig — COI amplicons show a band around 700bp. A 100bp ladder was used and the positive (700bp band) and negative control (no band) are shown. (TIFF) [file pntd.0007667.s004.TIFF]
