# Supplementary material for: Ecology and seasonality of sandflies and potential reservoirs of cutaneous leishmaniasis in Ochollo, a hotspot in southern Ethiopia
Source: PLoS Negl Trop Dis. 2019 Aug 19;13(8):e0007667. doi: 10.1371/journal.pntd.0007667 (PMC6715250; doi:10.1371/journal.pntd.0007667)
Supplement: S1 Table — The total number of sandflies, males and females caught per month. The number of not tested and tested females per month. The percentage of tested females, the number of kinetoplast DNA (kDNA) positive females and the kDNA prevalence (%) per month. (PDF) [file pntd.0007667.s005.pdf]

Month	Location type	# sample sites	# total sandflies	# female sandflies	# tested sandflies (%)	# tested kDNA+ sandflies (%)	Mean T (°C)	Mean %RH
**Mar '17**	Cave	3	653	279	274 (98%)	15 (5.47%)	21.94	49.22
	Rocky	3	152	67	67 (100%)	0 (0%)	23.17	47.72
	Stone fence	2	424	181	181 (100%)	0 (0%)	22.72	47.86
	Overall	8	1229	527	522 (99%)	15 (2.87%)	22.61	48.32
**Apr '17**	Cave	3	752	341	341 (100%)	11 (3.23%)	21.48	55.43
	Rocky	2	388	202	200 (99%)	5 (2.50%)	21.72	57.94
	Stone fence	3	855	469	468 (100%)	0 (0%)	22.24	53.10
	Overall	8	1995	1012	1009 (100%)	16 (1.59%)	21.81	55.79
**May '17**	Cave	4	907	348	343 (99%)	15 (4.37%)	18.14	84.31
	Rocky	2	117	28	28 (100%)	0 (0%)	18.93	83.49
	Stone fence	2	326	121	118 (98%)	2 (1.69%)	23.74	66.56
	Overall	8	1350	497	489 (98%)	17 (3.48%)	20.27	80.94
**Jun '17**	Cave	4	760	386	386 (100%)	9 (2.33%)	17.42	83.51
	Rocky	2	115	41	41 (100%)	0 (0%)	17.88	83.94
	Stone fence	2	229	119	119 (100%)	0 (0%)	20.06	70.37
	Overall	8	1104	546	546 (100%)	9 (1.65%)	18.45	81.53
**Jul '17**	Cave	4	650	319	284 (89%)	16 (5.53%)	16.70	87.33
	Rocky	2	173	92	92 (100%)	4 (3.53%)	17.12	89.37
	Stone fence	2	284	177	170 (96%)	6 (4.35%)	17.46	81.11
	Overall	8	1107	588	546 (93%)	26 (4.76%)	17.09	87.31
**Aug '17**	Cave	4	410	168	163 (97%)	14 (8.59%)	16.55	85.57
	Rocky	2	218	85	84 (99%)	1 (1.19%)	16.84	89.90
	Stone fence	2	151	74	74 (100%)	1 (1.35%)	17.61	77.90
	Overall	8	779	327	321 (98%)	16 (4.98%)	17.00	86.46
**Sep '17**	Cave	3	308	144	45 (31%)	1 (2.22%)	21.83	73.40
	Rocky	3	585	276	101 (37%)	0 (0%)	20.42	81.62
	Stone fence	2	213	143	43 (30%)	0 (0%)	20.60	74.35
	Overall	8	1106	563	189 (34%)	1 (0.53%)	20.95	77.06
**Oct '17**	Cave	4	350	173	167 (97%)	8 (4.79%)	20.39	74.56
	Rocky	2	371	187	184 (98%)	0 (0%)	21.43	72.47
	Stone fence	2	311	141	139 (99%)	0 (0%)	21.33	71.88
	Overall	8	1032	501	490 (98%)	8 (1.63%)	21.05	73.41
**Nov '17**	Cave	3	619	273	254 (93%)	12 (4.72%)	18.19	76.79
	Rocky	3	351	192	184 (96%)	7 (3.80%)	19.83	71.39
	Stone fence	2	293	160	155 (99%)	8 (5.16%)	19.72	70.93
	Overall	8	1263	625	593 (95%)	27 (4.55%)	19.25	74.01
**Dec '17**	Cave	3	575	310	284 (62%)	15 (5.28%)	21.79	56.28
	Rocky	3	401	255	244 (60%)	1 (0.41%)	21.32	64.04
	Stone fence	2	312	173	171 (73%)	1 (0.58%)	20.61	49.89
	Overall	8	1288	738	699 (65%)	17 (2.43%)	21.24	57.80
**Jan '18**	Cave	3	1344	606	373 (62%)	7 (1.88%)	20.59	44.22
	Rocky	2	286	126	75 (60%)	1 (1.33%)	24.30	52.60
	Stone fence	3	868	417	303 (73%)	4 (1.32%)	17.34	53.00
	Overall	8	2498	1149	751 (65%)	12 (1.60%)	20.74	48.51
**Feb '18**	Cave	3	790	397	345 (87%)	16 (4.64%)	22.32	37.53
	Rocky	2	492	226	208 (92%)	4 (1.92%)	24.17	43.85
	Stone fence	3	1157	714	637 (89%)	3 (0.47%)	20.19	42.06
	Overall	8	2439	1337	1190 (89%)	23 (1.93%)	22.23	40.24
**Year**	Cave		8118	3744	3259 (87%)	139 (4.27%)	19.92	67.35
	Rocky		3649	1777	1508 (85%)	25 (1.53%)	20.58	69.86
	Stone fence		5423	2889	2578 (89%)	23 (0.97%)	20.54	63.25
	Overall		17190	8410	7345 (87%)	187 (2.55%)	20.35	67.62

T=temperature

%RH = % relative humidity
