# Supplementary material for: Ecology and seasonality of sandflies and potential reservoirs of cutaneous leishmaniasis in Ochollo, a hotspot in southern Ethiopia
Source: PLoS Negl Trop Dis. 2019 Aug 19;13(8):e0007667. doi: 10.1371/journal.pntd.0007667 (PMC6715250; doi:10.1371/journal.pntd.0007667)
Supplement: S2 Table — For species identification, 200 sandflies were collection from July 2017 and again 200 from January 2018. Sample sites are the different places where sandfly traps were placed, which contribute together to the total collection in a particular habitat. ‘Collection’ describes the number of sandflies from a particular habitat or trap site that contributed to the total amount of sandflies collected during that month. This same proportion (percentages) was used for the sandflies that were brought to species level, here referred to as ‘selection’. (PDF) [file pntd.0007667.s006.pdf]

	July 2017											
**Habitat**	**Cave**				**Rocky area**			**Stone fence**				**Total**
**Collection / selection (% Total)**	650 / 118 (59%)				173 / 30 (16%)			284 / 52 (26%)				1107 / 200
**Sample site**	**C2**	**C3**	**C5**	**C9**	**R8**	**R16**	**R17**	**S9**	**S12**	**S16**	**S17**	
**Collection / selection**	243 / 43	219 / 40	66 /12	122 / 23	68 / 11	-	105 / 19	133 / 28	151 / 24	-	-	
**% (sample site/habitat)**	37%	34%	10%	19%	40%	-	60%	47%	53%	-	-	
	**January 2018**											
**Habitat**	**Cave**				**Rocky area**			**Stone fence**				**Total**
**Collection / selection**	1344 / 124 (62%)				286 / 26 (13%)			540 / 50 (25%)				2170 / 200
**Sample site**	**C2**	**C3**	**C5**	**C9**	**R8**	**R16**	**R17**	**S9**	**S12**	**S16**	**S17**	
**Collection / selection**	550 / 51	273 / 25	521 / 48	-	-	204 / 18	82 / 8	-	296 / 27	27 / 3	217 / 20	
**% (sample site/habitat)**	41%	20%	39%	-	-	71%	29%	-	55%	5%	40%	
